# Assessing the accuracy of automatic speech recognition for psychotherapy

**DOI:** 10.1038/s41746-020-0285-8

**Published:** 2020-06-03

**Authors:** Adam S. Miner, Albert Haque, Jason A. Fries, Scott L. Fleming, Denise E. Wilfley, G. Terence Wilson, Arnold Milstein, Dan Jurafsky, Bruce A. Arnow, W. Stewart Agras, Li Fei-Fei, Nigam H. Shah

**Affiliations:** 1 Department of Psychiatry and Behavioral Sciences, Stanford University, Stanford, CA USA; 2 Department of Health Research and Policy, Stanford University, CA, USA; 3 Center for Biomedical Informatics Research, Stanford University, Stanford, CA USA; 4 Department of Computer Science, Stanford University, Stanford, CA USA; 5 Department of Biomedical Data Science, Stanford University, Stanford, CA USA; 6 Departments of Psychiatry, Medicine, Pediatrics, and Psychological & Brain Sciences, Washington University in St. Louis, St. Louis, MO USA; 7 Graduate School of Applied and Professional Psychology, Rutgers, the State University of New Jersey, New Brunswick, New Jersey USA; 8 Clinical Excellence Research Center, Stanford University, Stanford, CA USA; 9 Department of Linguistics, Stanford University, Stanford, CA USA

**Keywords:** Translational research, Depression

## Abstract

Accurate transcription of audio recordings in psychotherapy would improve therapy effectiveness, clinician training, and safety monitoring. Although automatic speech recognition software is commercially available, its accuracy in mental health settings has not been well described. It is unclear which metrics and thresholds are appropriate for different clinical use cases, which may range from population descriptions to individual safety monitoring. Here we show that automatic speech recognition is feasible in psychotherapy, but further improvements in accuracy are needed before widespread use. Our HIPAA-compliant automatic speech recognition system demonstrated a transcription word error rate of 25%. For depression-related utterances, sensitivity was 80% and positive predictive value was 83%. For clinician-identified harm-related sentences, the word error rate was 34%. These results suggest that automatic speech recognition may support understanding of language patterns and subgroup variation in existing treatments but may not be ready for individual-level safety surveillance.

## Introduction

Although psychotherapy has proven effective at treating a range of mental health disorders, we have limited insight into the relationship between the structure and linguistic content of therapy sessions and patient outcomes^1–6^. This gap in knowledge limits insights into causal mechanisms of patient improvement, the evaluation and refinement of treatments, and the training of future clinicians^7^. Many patient and therapist factors have been assessed in psychotherapy (e.g., patient diagnosis, therapist experience, and theoretical orientation). However, there is little consensus as to which specific therapist behaviors contribute to patients’ symptom improvement or deterioration^2^.

Understanding what patients and therapists say during therapy, in conjunction with pre- and post-symptom assessment, may surface markers of good psychotherapy. Psychotherapy transcripts have long been used to search for objective, reproducible characteristics of effective therapists^8^. Also, analysis of psychotherapy transcripts has been used to generate theories and test hypotheses of specific mechanisms of action, but has been limited in part by technological capacity^9–11^. Discourse analysis is not common in controlled trials or effectiveness studies, and psychotherapy is rarely recorded outside of training settings or clinical trials. When it is recorded, a transcription is typically completed by a person, after which qualitative or quantitative analyses are undertaken. Manual transcription is expensive and time consuming^12^, leaving most psychotherapy unscrutinized^3^.

Automatic speech recognition (ASR) is being explored to augment clinical documentation and clinician interventions^3,13^. Evaluations of medical ASR systems often focus on individual dictation rather than modeling conversational discourse^14^, which is far more complex^15,16^. Prior literature estimates the word error rate of conversational medical ASR systems between 18 and 63%^17,18^. Although patient language analysis can inform diagnosis^19^, and clinician language use can inform treatment evaluation^12,20^, few approaches exist for transcribing clinical therapy sessions en masse. Although potentially useful, the need to audit emerging machine-learning systems has been highlighted by research showing that many ASR systems have worse performance for ethnic minorities^21^. Given existing health disparities in mental health treatment, there is a need to redress, rather than intensify equitable treatment across diverse groups^22,23^. Thus, methods to assess the performance of ASR systems in the mental health domain are needed.

In this work, we present an assessment of ASR performance in psychotherapy discourse. Using a sample of patient-therapist audio recordings collected as part of a US-based clinical trial^24^, we compare transcriptions generated by humans, which we consider the reference standard, to transcriptions generated by a commercial, cloud-based ASR service (Google Cloud Speech-to-Text)^25^. We quantify errors using three approaches. First, we analyze ASR performance using standard, domain-agnostic evaluation metrics such as word error rate. Second, we analyze patient symptom-focused language using a metric derived from a common depression symptom reporting tool, the Patient Health Questionnaire (PHQ-9)^26^. Third, we identify individual crisis moments related to self-harm and harm to others, and evaluate ASR’s performance in identifying these moments. Our evaluation, which uses a scalable HIPAA-compliant workflow for analyzing patient recordings, lays the foundation for future work using computational methods to analyze psychotherapy.

## Results

The study used a total of 100 therapy sessions between April 2013 and December 2016 containing 100 unique patients and 78 unique therapists. Among 100 patients for whom age was available (91%), the average age was 23 years (median 21; range, 18–52; SD, 5). A total of 87% of patients were female (Table 1). The average therapy session was 45 min (median, 47; range, 13–69; SD, 11) in length. During a session, the therapist spoke an average of 2909 words (median, 2,886; range, 547–6,213; SD, 1,128) over 20 min (median, 19; range, 4–41; SD, 8). The patient spoke an average of 3,665 words (median, 3,555; range, 277–7,043; SD, 1,550) over 25 min (median, 25; range 2–46; SD, 9). To characterize ASR in psychotherapy, a three-pronged evaluation framework is used: domain agnostic performance, depression symptom-specific performance, and harm-related performance.

**Table 1:** Patient demographics and therapy session information.

Patient demographics	Average	Standard deviation	Median	Min	Max
Number of patients	100	–	–	–	–
Female (%)	87	–	–	–	–
Age (years)	23	5	21	18	52
Session information					
Length					
Minutes	45	11	47	13	69
Number of words	6574	2102	6387	824	11,310
Time talking per session (min)					
Patient	25	9	26	2	46
Therapist	20	8	19	4	41
Words spoken per session (*n*)					
Patient	3665	1550	3555	277	7043
Therapist	2909	1128	2886	547	6213

### Domain agnostic performance

The first prong of our evaluation is domain agnostic, which uses word error rate and semantic distance to determine errors. The average word error rate of the speech recognition system was 25% (median, 24%; range, 8–74%; SD, 12%) (Table 2). Semantic distance is a proxy for the similarity of meaning between two sentences, based on computing a vector representation for the words in each sentence and looking at the distance between these vectors in Euclidean space^27^. The average semantic distance between human-transcribed and ASR-transcribed sentences was 1.2 points (median, 1.1; range, 0.5–2.4; SD, 0.3). For reference, the semantic distance between random words, random sentences, and human-selected paraphrases is 4.14, 2.97, and 1.14, respectively (Supplementary Tables 1 and 2).

**Table 2:** Similarity between the human-transcribed reference standard and ASR-transcribed sentences.

		Word overlap			Semantic similarity		
Group	*n*	Error Rate, %	Shapiro–Wilk	*p* value	Semantic distance, pts	Shapiro–Wilk	*p* value
Aggregate							
Total	100	25% ± 12%	0.93	<0.001	1.20 ± 0.31	0.97	0.03
Speaker							
Patient	100	25% ± 12%	0.86	<0.001	1.19 ± 0.33	0.94	<0.001
Therapist	100	26% ± 11%	0.88	<0.001	1.20 ± 0.29	0.99	0.57
Patient gender							
Male	13	24% ± 9%	0.95	0.55	1.17 ± 0.30	0.95	0.55
Female	87	25% ± 13%	0.84	<0.001	1.19 ± 0.33	0.94	<0.001

Plus/minus values denote standard deviation. Lower error rate is better. Lower semantic distance is better. Shapiro–Wilk tests were conducted to test the normality assumption (Supplementary Fig. 2). Low *p* values indicate the data are not normally distributed.

**Fig. 1:**
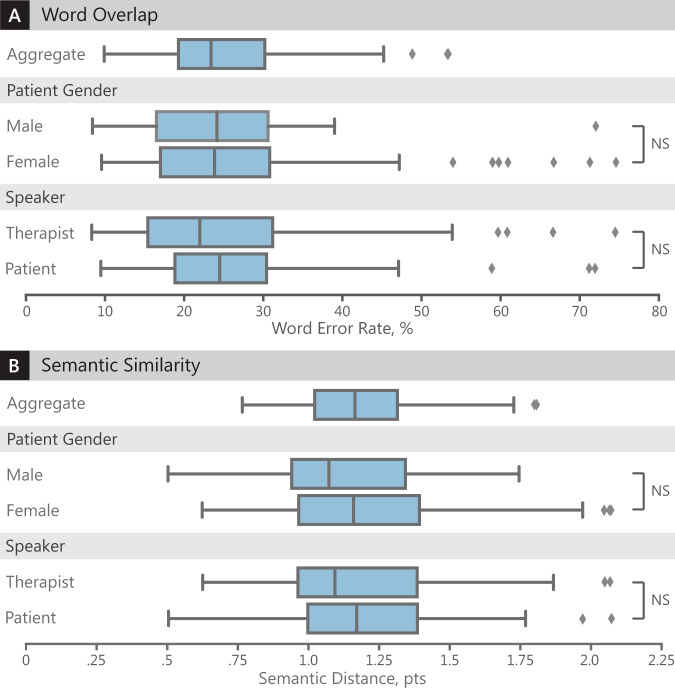
Automatic speech recognition performance, overall and by subgroup. Evaluation of ASR transcription performance compared to the human-generated reference transcription. Each box denotes the 25th and 75th percentile. Box center-lines denote the median. Whiskers denote the minimum and maximum values, excluding any outliers. Outliers, denoted by diamonds, are defined as any point further than 1.5× the interquartile range from the 25th or 75th percentile. Sample sizes are listed in Table 2. NS not significant means the difference is not statistically significant. **a** Comparison of word overlap (i.e., word error rate). Lower word error rate is better. **b** Comparison of semantic similarity (i.e., semantic distance). Lower semantic distance is better.

Transcription of patients’ speech was not significantly different from therapists’ speech (25% vs 26% error rate, two-tailed Mann–Whitney U-test, *p* = 0.21) (Fig. 1). In addition, transcription of male speech was not significantly different from female speech (24% vs 25% error rate, two-tailed Welch’s *t*-test, *p* = 0.69).

### Depression symptom specific performance

The second prong of our evaluation is depression-specific. Across medical terms from the Patient Health Questionnaire^26^, the average sensitivity (i.e., recall) was 80% and positive predictive value (i.e., precision) was 83% (Table 3). The PHQ category with the highest sensitivity was category 2 (depression) with a sensitivity of 85%. The categories with the highest positive predictive value were categories 5 (overeating) and 7 (mindfulness) with a positive predictive value of 100%. Results are presented for each medical term in Supplementary Table 3.

**Table 3:** Performance on clinically-relevant utterances by patients.

PHQ	Keywords^a^	Number of positives	True positives	False negatives	False positives	Sensitivity	Positive predictive value
1	Interest, interested, interesting, interests, pleasure	169	127	42	38	75%	77%
2	Depressed, depressing, feeling down, hopeless, miserable	74	63	11	12	85%	84%
3	Asleep, drowsy, sleepiness, sleeping, sleepy	114	85	29	19	75%	82%
4	Energy, tired	143	115	28	22	80%	84%
5	Overeat, overeating	5	3	2	0	60%	100%
6	Bad, badly, poorly	405	336	69	56	83%	86%
7	Mindfulness	11	9	2	0	82%	100%
8	Fidget, fidgety, restless, slow, slowing, slowly	39	28	11	13	72%	68%
9	Dead, death, depression, died, suicide	103	86	17	18	83%	83%
	Weighted average	1063	852	211	178	80%	83%

^a^For each question of the Patient Health Questionnaire (PHQ-9), relevant keywords were identified by querying the Unified Medical Language System using each PHQ question to generate search terms. Each table row denotes a different question from the PHQ-9. Number of occurrences refer to how often the keywords appear in our transcribed therapy sessions. True positives refer to a correct transcription by the automatic speech recognition system. False negatives and false positives denote incorrect transcriptions. Sample size is denoted by the number of positives.

### Harm-related performance

The third prong of our evaluation centers on harm-related performance. A total of 97 clinician-identified harm-related sentences were identified. Half of the manually annotated sessions (50%; 10 of 20) had at least one harm-related utterance. These sentences demonstrated an average error rate of 34% (median, 16%; range 0–100%; SD, 37%) and average semantic distance of 0.61 (median, 0.30; range 0–2.62; SD, 0.75). Compared with performance across all therapy sentences, harm-related sentences demonstrated a higher word error rate (34% vs 25% error rate, two-tailed Mann–Whitney *U*-test, *p* = 0.07) but a significantly lower semantic distance (0.61 vs 1.20, two-tailed Mann–Whitney *U*-test, *p* < 0.001).

For the 45 harm-related sentences spoken by the therapist, the average error rate was 36% (median, 20%; range, 0–100%; SD, 39%). For the 52 harm-related sentences spoken by the patient, the average error rate was 32% (median, 13%; range 0–100%; SD, 35%). Sentences spoken by the patient were not significantly different from sentences spoken by the therapist in terms of word error rate (32% vs 36%, two-tailed Mann–Whitney *U*-test, *p* = 0.60) and semantic distance (0.62 vs 0.58, two-tailed Mann–Whitney *U*-test, *p* = 0.59). Table 4 illustrates the importance of semantic distance, in the context of transcription errors. Several sentences are categorized by the type of their transcription error, thus demonstrating the clinical relevance of surface differences in words, or phonetics, versus deeper semantic errors.

**Table 4:** Transcription errors made by the automatic speech recognition system.

		Meaning (semantics)	
		Similar to reference standard	Different from reference standard
Form (Words or Phonetics)	Similar to reference standard	1. Tuesday, I had found out about that my grandmother had died is dying. 2. Came back and ate eat some more.	1. I have still been feeling depressed the preston. 2. Do you have any plans to hurt dirt yourself?
	Different from reference standard	1. Depends on like what I eat or what I’ve been eating have been made. 2. Comfortable to expressing his these negative emotions.	1. It still stings. It doesn’t hurt as much as it did wasn’t hers do you still feel like. 2. I’m going to try to appeal kill the schools.

Each numbered sentence is a different sentence containing both the reference standard and ASR transcription. Strikethrough denotes the human-generated reference standard. Underline denotes the speech recognition system’s erroneous output. Black text denotes agreement.

## Discussion

We proposed the use of semantic distance, clinical terminology, and clinician-labeled utterances to better quantify ASR performance in psychotherapy. This is more comprehensive than word error metrics alone, which treat all errors (e.g., word substitutions, additions) as equal. Our evaluation found a general error rate of 25%, which varied by use case (e.g., symptom detection vs harm-related utterances). When evaluated using semantic similarity and not error rate, the ASR system was significantly better at transcribing clinician-labeled sentences related to harm than other sentences spoken during the session. This suggests that acceptable performance may vary depending on clinical use case and choice of evaluation framework.

Given these findings, using ASR to passively collect symptom information may be possible, as currently only 20% of mental health practitioners use measurement-based care^28^. Creating transcripts is important because their inspectability offers a benefit for clinician training and supervision compared to using black-box deep learning models^29,30^, which may have predictive validity, but are challenging to interpret^31^. However, critical words used to diagnose depression had different rates of performance (Table 3), ranging from 60 to 100%. More research is warranted in symptom-focused accuracy, as culturally sensitive diagnostic accuracy will be crucial if ASR is to aid in clinical documentation. Special attention to algorithmic performance is especially crucial in healthcare settings to ensure equitable performance across patient and provider subgroups (e.g. age, race, ethnicity, gender, diagnosis)^32–34^. Although ASR is unlikely to be first used to detect harm-related utterances in clinical settings, assessing risk of harm to self or others is a cornerstone of clinician duty. Thus, recognizing harm-related phrases is crucial to any downstream processes and merits special attention.

A known bottleneck in psychotherapy research is that psychotherapy sessions are rarely examined in their entirety, which impedes analysis of practice patterns^35^. Despite assumptions of provider uniformity in randomized clinical trials and naturalistic investigations^36,37^, therapist effects–that some therapists consistently achieve better results than others–is well documented^38,39^. Accurate transcriptions would facilitate more rigorous quality assessment than is currently feasible^6,40^. ASR provides a potential avenue to study such effects using computational approaches.

Although ASR is not perfect, it may enable better therapist training. For instance, ASR may quickly surface illustrations of patient idioms of distress^41^, or effective examples of appropriate and inappropriate clinician responses. Similarly, ASR-generated transcripts could aid in linking speech acts to theoretically important phenomenon such as therapeutic alliance, the most consistent predictor of psychotherapeutic outcome^42^. Although these applications may seem distant, a more proximal application of this technology could be to facilitate the supervision of trainees, in which licensed clinicians review trainees’ transcripts. ASR can accelerate this process, however, integrating ASR into clinical practice will require thoughtful design and implementation^6^. Additional use cases of ASR in medicine extend to patient symptom documentation^13,18^, exploring communication-based ethnic disparities in treatment^40,43,44^, assessing dissemination efforts of evidence-based practices^45,46^, pooling, and standardizing transcripts from psychotherapy studies^40^, and monitoring harmful or illegal clinician behavior^47^.

Our work has limitations. First, we analyzed ASR performance on outpatient psychotherapy sessions between therapists and college-aged participants. These results may not generalize to other patient or provider populations^48^. Second, our evaluation uses transcriptionist-generated timestamps for each spoken phrase. These transcriptionists may provide inaccurate timestamps due to delayed reaction times or other human errors. Third, to maximize reproducibility, we limit our analysis to words directly from the PHQ-9 and Unified Medical Language System (Table 3)^49^. These lists are not meant to be exhaustive, and future research should seek to expand this list to additional clinically-relevant terminology^50–56^. Fourth, while our evaluation method analyzed ASR performance broken down by the role of patient versus therapist, such role annotations were only available in the human-annotated transcriptions. It is unknown how well ASR performs role assignment (i.e., speaker diarization). Fifth, it is possible that the human-generated transcripts had inaccuracies. As a result, our estimates are likely conservative. Sixth, we note that while we did choose a state-of-the-art tool for automatic transcription, other ASR systems may perform differently^21^. Assessing transcription accuracy across tools and clinical settings is a crucial next step^21^. Seventh, we use one method for computing word embeddings (Word2Vec^27^) and sentence embeddings (earth mover distance^57^) to establish this baseline, however other appropriate options exist and should be assessed in future work (e.g., BioBERT, GloVe)^58–60^. However, by establishing a three-pronged evaluation framework, we enable a more nuanced comparison of ASR systems than currently allowed by word error rate-based approaches.

ASR will likely be useful before it is perfect. Thus, it is crucial to design evaluations that differentiate between the types of errors, assess clinical impact, and detail performance for legally mandated situations such as self-harm^61,62^. ASR holds promise to convert psychotherapy sessions into computable data at scale; and with enough data, characteristics of effective therapy may be uncovered via supervised machine learning and discourse analysis. However, claims regarding the potential of artificial intelligence should be tempered in the context of real performance metrics, and challenges in fairness, maintaining privacy, and trust^63–66^. ASR may offer a cost-effective and reproducible way to transcribe sensitive conversations, but collecting and analyzing intimate data at unprecedented scales demands improved governance around limiting unintended use and tracking provenance of the conclusions drawn^67–75^.

The National Institute of Mental Health has called for computational approaches to understand trajectories of mental illness and to create standardized data elements^76^. With improved accuracy and the development of agreed-upon thresholds for acceptable performance, mechanisms of action in psychotherapy would be easier to uncover. Our work, which uses a scalable, HIPAA-compliant workflow for analyzing patient recordings, lays the foundation for future work using computational methods to analyze psychotherapy. By facilitating better descriptions of psychotherapeutic encounters associated with good outcomes, ASR can help illuminate precise interventions that improve psychotherapy effectiveness and allow us to revisit long-held ideas of psychotherapy with more objective, inspectable, and scalable analyses.

In conclusion, we outlined a three-pronged evaluation framework spanning domain agnostic performance, clinical terminology, and clinician-identified phrases to characterize ASR performance in psychotherapy. Compared to human-generated transcripts, ASR software demonstrated a word error rate of 25% and a mean semantic distance of 1.2, which is likely sufficient to enable research aimed at understanding existing treatments and to augment clinician training. However, accuracy, in terms of word error rate and semantic distance, varied for depression-related words and for harm-related phrases, suggesting a need for both improved accuracy and the development of agreed-upon thresholds for use in safety monitoring. ASR can potentially enable psychotherapy effectiveness research but requires further improvement before use in safety monitoring. Our work lays the foundation for using computational methods to analyze psychotherapy at scale.

## Methods

### Study design

This study is a secondary analysis of audio recordings of 100 therapy sessions from a cluster randomized trial. Audio recordings of college counseling psychotherapy were gathered per protocol during the trial, which had a primary aim of studying two clinician training strategies^24^. Written consent was obtained per protocol in the original trial from both patients and therapists. The primary objective of the current study is to quantify the accuracy of automatic speech recognition software via a comparison with the human-generated transcripts on overall accuracy, depression-specific language, and harm-related conversations.

This study was conceptualized and executed after the design and launch of the original study. All research procedures for this study were reviewed and approved by the Institutional Review Board at Stanford University. During the original trial, all therapists were consented by Washington University in St. Louis, and all patients involved in the study were consented by their local institutions. The Stanford University Institutional Review Board approved all consent procedures. Although approaches will vary between organizations, we describe our process for establishing a HIPAA-compliant ASR process in Supplementary Note 1.

### Clinical setting and data collection

This study assessed audio recordings of 100 therapy sessions from 100 unique patient-therapist dyads. The sessions took place between April 2013 and December 2016 at 23 different college counseling sites across the United States. Audio recordings were collected in the original study for humans to review and assess therapist quality.

### Corpus creation

In order to compare the ASR to human-generated transcripts, two transcriptions were done: one using industry-standard manual transcription services, and the other using a commercially-available ASR software^25^. A third-party transcription company was paid to create the transcriptions by listening to the original audio. Scribes transcribed all words including “filler words” (e.g., -*huh-*, *-mm-hm-*). The protocol for manual transcription is provided in Supplementary Note 2. Each utterance was “diarized” (i.e., ascribed to a speaker: therapist, patient, or unknown) and each change of speaker was timestamped in minutes and seconds. The human-generated transcripts were used as the reference standard for all comparisons. Data storage, transmission, and access were assessed and approved by the Stanford University Information Security Office and the Stanford University Institutional Review Board.

### Measures of automatic speech recognition performance

There are currently no standard approaches to assessing ASR quality in psychotherapy. We propose three approaches: (1) a general, commonly used domain agnostic evaluation; (2) examining symptom-specific language; and (3) examining crucial phrases related to self-harm or harm to others.

*Domain agnostic evaluation measures:* The standard evaluation metric for speech recognition systems is word error rate (WER)^77,78^, defined as the total number of word substitutions (S), deletions (D), and insertions (I) in the transcribed sentences, divided by the total number of words (N) in the reference sentence (i.e., human-transcription). That is, WER = (S + D + I)/N. The word error rate requires an exact word match to be considered correct. Homophones (i.e., words that sound the same but have different meanings like “buy” and “bye”) were measured as inaccuracies.

One shortcoming of word error rate is how it assigns equal importance to all words. Transcribing the word “death” into “dead” will be registered as an error. However, such an error may not significantly change the meaning of the sentence and in fact may be sufficiently correct for clinical use. This can be partly mitigated by using relative word importance to re-weight the final metric accordingly^79,80^. However, this still measures word-level equivalence rather than sentence-level resemblance^81^.

To address these shortcomings, we propose measuring semantic distance between each ASR-generated transcription and human-generated transcript. While subjective measures of semantic similarity for machine translation and paraphrase detection exist^82–85^, large-scale manual review by humans is generally infeasible. Therefore, we used word2vec embeddings^27^ to extract word-level embeddings followed by mean-pooling to compute a sentence-level embedding^86^. The sentence embeddings of the human-generated transcripts were compared to the ASR-generated embeddings using earth mover distance^57^. A comparison of earth mover and cosine distance is shown in Supplementary Fig. 1. A smaller value of semantic distance indicates higher similarity, with zero semantic distance indicating perfect similarity.

*Depression-specific evaluation:* Assessing domain-specific vocabulary in health contexts has been called for by researchers from the Centers for Disease Control and Prevention and the U.S. Food and Drug Administration^87^. To evaluate depression-specific vocabulary, we selected clinically-relevant words directly from a commonly used depression screen, the Patient Health Questionnaire (PHQ-9)^26^. Keywords from the PHQ-9 (e.g., sleep, mood, suicide) were extended to a larger list using the Unified Medical Language System, a medical terminology system maintained by the U.S. National Library of Medicine^88^. This is similar to previous approaches used to search for medical subdomain language^89^. While there are methods to expand the vocabulary to synonyms and informal phrases^90^, in this work, our goal was to provide a baseline that allows for simplicity and reproducibility^87^. Our approach using the Unified Medical Language System was selected to prioritize false negatives (Type II errors) over false positives (Type I errors) for symptom detection. This approach may differ across use cases.

Once the list of clinically-relevant words was determined, sensitivity and positive predictive values were computed from the perspective of binary classification. Clinically-relevant words were treated as positive examples and all other words were treated as negative examples. For each clinical word, transcription performance was measured across all therapy sessions. For each word (positive example), the number of negative examples is large, consisting of the set of every other word in the English language, thus leading to very high specificity rates (i.e., above 99.9%). Because it would not meaningfully differentiate performance, we do not report specificity.

*Harm-related evaluation:* A licensed clinical psychologist (Author: A.S.M.) randomly sampled and retrospectively read 20 transcripts from the dataset and annotated any harm-related phrases spoken by the patient or therapist (e.g., “I want to hurt myself”). The harm-related sentences are a subset of the full dataset in Table 1. We then assessed the accuracy of ASR on this subset. This assessment was of historical data, and thus no safety concerns were shared with law enforcement or other mandated reporting agencies.

### Statistical analyses

Before testing for a difference of means, subgroups were tested against the normality assumption and their variance was assessed. To test the normality assumption, the Shapiro–Wilk test was used (Supplementary Fig. 2). To test for equal subgroup variance, the Levene test was used. Depending on the Shapiro–Wilk and Levene test results, one of the following difference tests were used: two-tailed Welch’s t-test or two-tailed Mann–Whitney *U*-test. The significance threshold was *p* = 0.01. All statistical analyses were implemented in Python (version 3.7; Python Software Foundation) with the SciPy software library^91^. Covariates were the word error rate and semantic distance.

### Reporting summary

Further information on research design is available in the Nature Research Reporting Summary linked to this article.

## Supplementary information

Supplemental material

Reporting summary

## Data Availability

The dataset is not publicly available due to patient privacy restrictions, but may be available from the corresponding author on reasonable request.

## Code availability

The code used in this study can be found at:https://github.com/som-shahlab/psych-audio.
