# Supplementary material for: Assessing the accuracy of automatic speech recognition for psychotherapy
Source: NPJ Digit Med. 2020 Jun 3;3:82. doi: 10.1038/s41746-020-0285-8 (PMC7270106; doi:10.1038/s41746-020-0285-8)
Supplement: Supplementary file 1 — Supplemental material [file 41746_2020_285_MOESM1_ESM.pdf]

Assessing the Accuracy of Automatic Speech Recognition for Psychotherapy

Supplementary Information

Contents

A. Supplementary Tables 2

Supplementary Table 1. Sample sentence pairs 2

Supplementary Table 2. Similarity of different sentence pairs 2

Supplementary Table 3. Performance grouped by clinically-relevant utterances spoken by the patient 3

B. Supplementary Figures 4

Supplementary Figure 1. Comparison of semantic distance metrics 4

Supplementary Figure 2. Shapiro-Wilk tests for normality 4

Supplementary Figure 2 (continued). Shapiro-Wilk tests for normality 5

C. Supplementary Notes 6

Supplementary Note 1: HIPAA-Compliant Implementation 6

Supplementary Note 2: Human Transcription Guide 7

D. Supplementary References 7

A. SUPPLEMENTARY TABLES

Supplementary Table 1. Sample sentence pairs

Relationship	Sentence/Phrase 1	Sentence/Phrase 2
Random words	Atone denotations continuations	Carpet hired cheesecakes
Random sentences	Arises from the fact	Yes, I think we did
Paraphrases	The committee recommends to	The board recommended that
ASR transcription	It doesn't hurt as much as it did	It wasn't hers do you still feel like
Perfect transcription	I have still been feeling depressed	I have still been feeling depressed

Word error rate and earth mover distance are computed on a pair of sentences. Each sentence in this pair is generated in one of several ways. Random words are incomprehensible sentences consisting of random words. Random sentences are real, English sentences from the PPDB dataset[1]. However, each sentence is unrelated to one another. PPDB paraphrases are pairs of sentences that are considered paraphrases, as determined by human judgment. ASR transcription denotes sentence pairs from our psychotherapy dataset, where one sentence is the human-transcribed reference-standard sentence and the second sentence is the output from the ASR system. Perfect transcription denotes the same sentence.
PPDB paraphrase database[1], *ASR* automatic speech recognition

Supplementary Table 2. Similarity of different sentence pairs

	Word Overlap (WER, %)				Semantic Similarity (EMD, pts)			
	Mean	SD	Median	Range	Mean	SD	Median	Range
Random words	100	0.1	100	93-100	4.14	0.22	4.14	3.03-5.80
Random sentences	98	9	100	25-100	2.97	0.53	2.98	0.85-5.03
Paraphrases	48	23	50	17-100	1.14	0.51	1.05	0.19-3.86
ASR transcription	25	12	24	8-74	1.20	0.31	1.1	0.5-2.4
Perfect transcription	0	0	0	0-0	0	0	0	0-0

WER word error rate (lower is better), *EMD* earth mover distance (lower is better), *SD* standard deviation, *ASR* automatic speech recognition

Supplementary Table 3. Performance grouped by clinically-relevant utterances spoken by the patient

PHQ Question	Keyword	Occurrences	True Positives	False Positives	False Negatives	Sensitivity	Positive Predictive Value
1	interesting	52	43	9	9	83%	83%
1	interested	23	18	4	5	78%	82%
1	interest	8	7	9	1	88%	44%
1	interests	5	3	1	2	60%	75%
1	pleasure	2	2	3	0	100%	40%
2	depressed	24	19	7	5	79%	73%
2	miserable	13	12	0	1	92%	100%
2	hopeless	2	1	1	1	50%	50%
2	depressing	2	2	0	0	100%	100%
2	feeling down	2	1	3	1	50%	25%
3	sleeping	43	33	8	10	77%	80%
3	asleep	27	23	6	4	85%	79%
3	sleepy	3	2	1	1	67%	67%
3	sleepiness	1	1	0	0	100%	100%
4	tired	65	51	12	14	78%	81%
4	energy	24	21	4	3	88%	84%
5	overeat	2	2	0	0	100%	100%
6	bad	212	175	32	37	83%	85%
6	badly	6	3	3	3	50%	50%
6	poorly	5	4	0	1	80%	100%
7	mindfulness	5	5	0	0	100%	100%
8	slow	10	6	3	4	60%	67%
8	slowly	7	5	7	2	71%	42%
8	fidgety	5	5	1	0	100%	83%
8	restless	4	3	1	1	75%	75%
8	slowing	2	1	0	1	50%	100%
8	fidget	1	0	0	1	0%	-
9	depression	40	36	4	4	90%	90%
9	died	16	13	3	3	81%	81%
9	dead	11	9	4	2	82%	69%
9	death	6	3	2	3	50%	60%
9	suicide	3	2	0	1	67%	100%

PHQ patient health questionnaire. Higher sensitivity and positive predictive value is better.

B. SUPPLEMENTARY FIGURES

Supplementary Figure 1. Comparison of semantic distance metrics

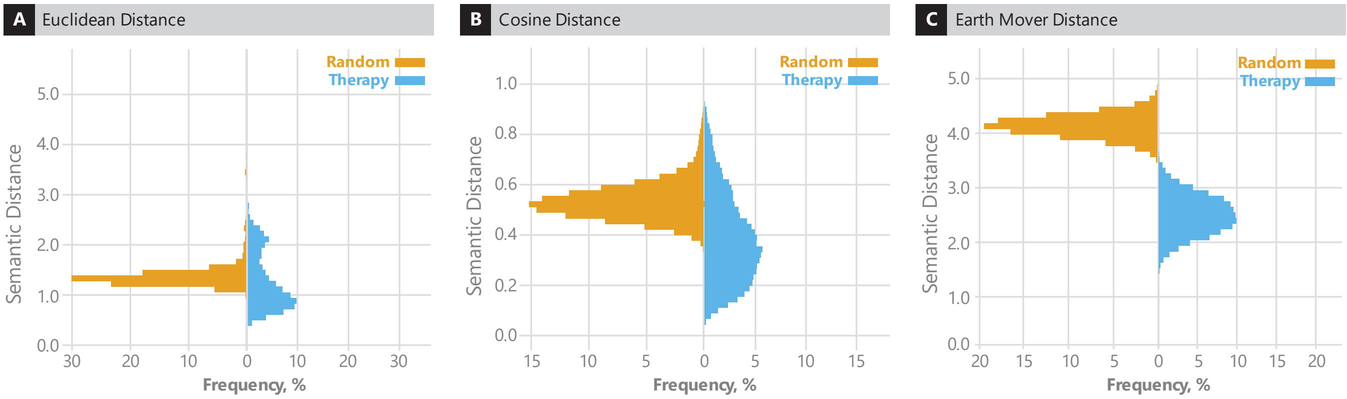

First, 1,000 sentences were selected from the human-transcribed sentences (ie, corpus). Second, sentence embeddings were computed for each sentence using word2vec. Third, the pairwise distance was computed between the 1,000 sentence embeddings. Finally, a histogram of distances was created and plotted. This process was repeated with completely random sentences (ie, sentences of random length). These sentences contained uniformly random English words selected from the file /usr/share/dict/words on a Linux (CentOS 7) computer.

Supplementary Figure 2. Shapiro-Wilk tests for normality

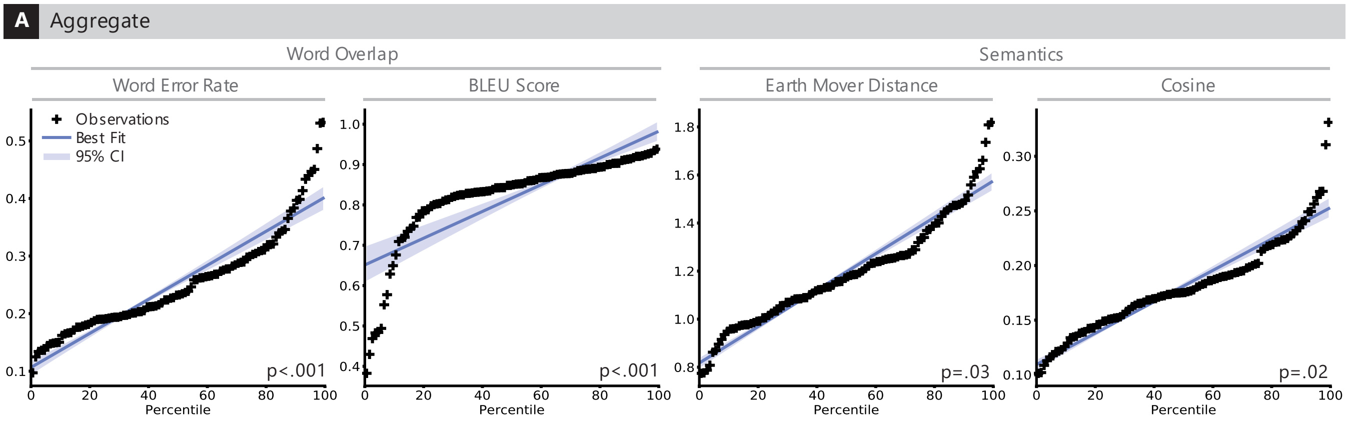

BLEU bilingual evaluation understudy[2]

The plots show a Shapiro-Wilk test and examine whether the histograms in Supplementary Figure 1 follow a normal distribution. At the bottom right of each plot, the p-value is listed. A perfect normal distribution would show black points exactly on the blue line. Black points further from the blue line indicate deviations from the theoretical normal distribution.

Supplementary Figure 2 (continued). Shapiro-Wilk tests for normality

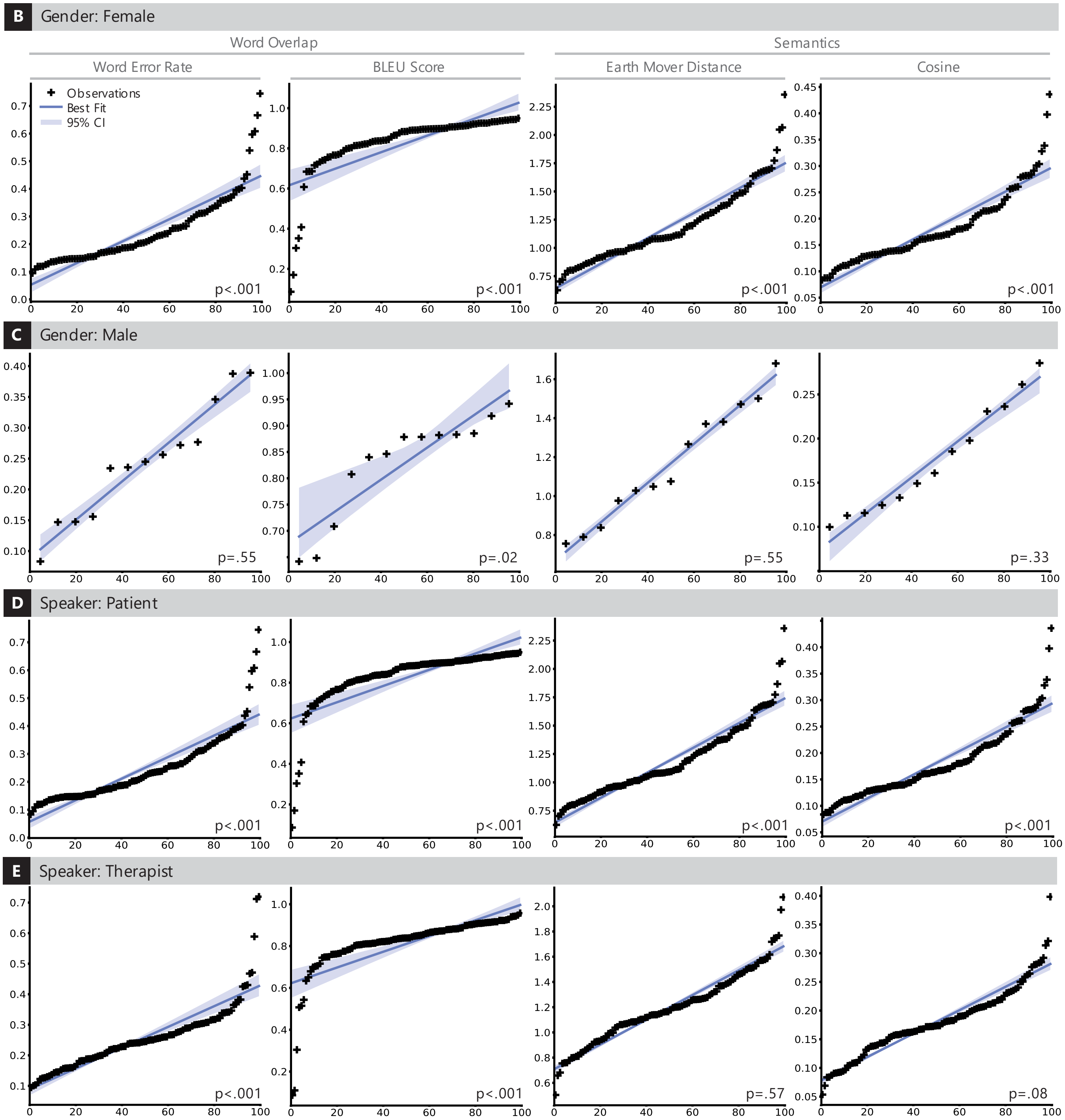

BLEU bilingual evaluation understudy[2]

The plots show a Shapiro-Wilk test and examine whether the histograms in Supplementary Figure 1 follow a normal distribution. At the bottom right of each plot, the p-value is listed. A perfect normal distribution would show black points exactly on the blue line. Black points further from the blue line indicate deviations from the theoretical normal distribution.

C. SUPPLEMENTARY NOTES

Supplementary Note 1: HIPAA-Compliant Implementation

Administrative. Our team determined which data elements constituted alone, or in combination with other data elements, protected health information (PHI), as defined by HIPAA. Audio recordings of individual patients are PHI. Thus, we coordinated with our IRB and Privacy office to ensure data storage, transmission, and access met institutional standards. This process took around 8 months at our institution. Although we used Google Cloud Speech-To-Text for automatic speech recognition, other commercial and open-source alternatives are available (eg, Microsoft, Amazon, Kaldi). At the time of the study, Stanford University had an existing Business Associates Agreement in place with Google, which is a federal requirement for sharing PHI. Implementation at sites without a BAA should expect a longer study duration.

Additional information available at:
U.S. Department of Health & Human Services. Retrieved from https://web.archive.org/web/20190619121056/https://www.hhs.gov/hipaa/for-professionals/privacy/special-topics/de-identification/index.html. Accessed June 21, 2019.

Technical. During the human transcription process, human annotators were instructed to anonymize mentions of specific places, names, or dates. These are reflected in the transcript using bracket delimiters and the type of word being omitted (eg, [PLACE] or [NAME]). The annotators did not provide specific timestamps for which words were omitted. The original audio recording was not scrubbed. As a result, the automatic speech recognition system may transcribe specific names, places, or dates.

Data was standardized in a two-step process. First, the human annotators transcribed sentences spoken in the audio recording. Each time a *talk turn* (ie, change of speaker) occurred, the annotator typed the transcribed text into a plain text file, one sentence per line. Each line contains the speaker (therapist or patient), the starting timestamp of the sentence (in minutes and seconds) and the actual transcribed text. Second, we further standardized the human transcriptions into a structured JSON format for easier algorithm ingestion. No PHI was stored in these human annotation, reference standard JSON files.

Audio recordings were uploaded from our HIPAA-compliant secure server at Stanford to a PHI-safe Google cloud folder called a *bucket.* The research team must access the secure Stanford server through Stanford-approved secure connections (ie, VPN). In addition to a strong password, the VPN requires two-factor authentication (eg, cell phone text message or push notification). Once connected to the Stanford VPN, to connect to the secure Server, an additional two-factor authentication step must be performed. This is because the Stanford VPN may be used for academic purposes outside the scope of this project.

Once the audio recordings were uploaded to the PHI-safe cloud bucket, we wrote a Python script on the secure Stanford server to execute a single Google Speech-to-Text API call, once per audio recording. Because each audio recording is 30 to 60 minutes in duration, each Speech-to-Text API call took between 10 and 15 minutes. The API call returns a JSON-like result to the Stanford server. This result contained the transcribed text and millisecond-level timestamps for each word, which were immediately saved onto the Stanford's server encrypted hard drives. At no point during this process did any audio files or transcription text files leave the secure Stanford server or PHI-safe Google cloud bucket.

Supplementary Note 2: Human Transcription Guide

Formatting Guidelines

- File should be in .txt file format
- The document should NOT use a table
- Each document should include the following metadata:
- Audio filename, transcript filename, therapist gender, patient gender
- Any names of people (first or last) should be replaced with: (name)
- Any mention of a city or a state should be replaced with: (location)
- Any mention of a phone number, address, or email should be replaced with (PHI)
- Follow normal capitalization practices (eg, uppercase state names), and punctuation included (eg, "?" , ",")
- Conjunctions should be transcribed as conjunctions (eg, "don't" should be "don't" rather than "do not"
- Colloquialisms should be written as true to the speaker's intent as possible, but with regular spelling. (eg, "cuz" should be spelled out as "because")
- Numbers and times should be transcribed with digits, rather than spelled out (ex. "4:30" should be "4:30" rather than "four thirty", and "57" should be "57" rather than "fifty seven")
- The words "um", "uh", "uh-huh", "uh-oh", "oh", "hmm" should be included
- Periods of laughter or crying should be marked by (laughing) or (crying)
- The timestamps denote when the given speaker began speaking and are formatted as (minutes:seconds) relative to the start of the audio file
- If it is ever unclear to the person transcribing whether the speaker of the therapist said some word or phrase, the symbol U should be used in place of T or P

Example Document Structure

Audio filename: 2019_07_01_Patient_4321_Site_12.mp3
Transcript filename: 2019_07_01_Patient_4321_Site_12.txt
Therapist (T) gender: Male / female / unknown
Patient (P) gender: Male / female / unknown

T (0:07): words that the therapist says
P (1:34): words that the patient says
U (1:58): words whose speaker is unknown

D. SUPPLEMENTARY REFERENCES

1. Pavlick, E., Rastogi, P., Ganitkevitch, J., Van Durme, B. & Callison-Burch, C. PPDB 2.0: Better paraphrase ranking, fine-grained entailment relations, word embeddings, and style classification. *Annual Meeting of the Association for Computational Linguistics* 425–430 (2015) doi:10.3115/v1/P15-2070
2. Papineni, K., Roukos, S., Ward, T. & Zhu, W-J. BLEU: a method for automatic evaluation of machine translation. *Annual Meeting of the Association for Computational Linguistics* 311–318 (2002) doi:10.3115/1073083.1073135
